# Aurora B-dependent phosphorylation of Ataxin-10 promotes the interaction between Ataxin-10 and Plk1 in cytokinesis

**DOI:** 10.1038/srep08360

**Published:** 2015-02-10

**Authors:** Jie Tian, Chuan Tian, Yuehe Ding, Zhe Li, Qizhi Geng, Zhikai Xiahou, Jue Wang, Wenya Hou, Ji Liao, Meng-Qiu Dong, Xingzhi Xu, Jing Li

**Affiliations:** 1 Beijing Key Laboratory of DNA Damage Response, College of Life Sciences, Capital Normal University, Beijing 100048, China; 2 National Institute of Biological Sciences, Beijing 102206, China

## Abstract

Spinocerebellar ataxia type 10 (SCA10) is an autosomal dominant neurologic disorder caused by ATTCT expansion in the *ATXN10* gene. Previous investigations have identified that depletion of Ataxin-10, the gene product, leads to cellular apoptosis and cytokinesis failure. Herein we identify the mitotic kinase Aurora B as an Ataxin-10 interacting partner. Aurora B interacts with and phosphorylates Ataxin-10 at S12, as evidenced by in vitro kinase and mass spectrometry analysis. Both endogenous and S12-phosphorylated Ataxin-10 localizes to the midbody during cytokinesis, and cytokinetic defects induced by inhibition of *ATXN10* expression is not rescued by the S12A mutant. Inhibition of Aurora B or expression of the S12A mutant renders reduced interaction between Ataxin-10 and polo-like kinase 1 (Plk1), a kinase previously identified to regulate Ataxin-10 in cytokinesis. Taken together, we propose a model that Aurora B phosphorylates Ataxin-10 at S12 to promote the interaction between Ataxin-10 and Plk1 in cytokinesis. These findings identify an Aurora B-dependent mechanism that implicates Ataxin-10 in cytokinesis.

Ataxin-10 is the disease-causing protein for Spinocerebellar ataxia type 10 (SCA10), the only disease known to be caused by pentanucleotide expansions. SCA10 is manifested by cerebellar ataxia and epilepsy^1^. While normal people have between 10–29 copies of the ATTCT pentanucleotide repeats in the intron 9 of the *ATXN10* gene, inflicted patients have between 800–4,500 repeats^2^,^3^. The aberrant ATTCT repeats do not interfere with gene transcription, since the expanded repeats are transcribed at normal levels in patient-derived cells and the pre-mRNA is processed normally^4^. White et al.^5^ showed that the expanded ATTCT repeats bind to and sequester hnRNP K, which results in decreased hnRNP K activity, leading to translocation of PKC δ to the mitochondria, caspase-3 activation and apoptosis. The toxic RNA mediated gain-of-function of the ATTCT repeats are also confirmed in studies of transgenic mice, which display motor phenotypes and susceptibility to seizure resembling those seen in SCA10 patients^6^.

*ATXN10* encodes the Ataxin-10 protein, whose function is largely unknown. Cytological studies using rat, mouse and human brain sections reveal that Ataxin-10 is predominantly cytoplasmic^7^,^8^. Ataxin-10 contains two armadillo repeats^7^, which might bind cytoskeleton^9^. Ataxin-10's binding partners include G-protein β2 subunit^8^ and *O*-linked β-N-Acetyleglucosamine transferase (OGT)^10^,^11^. It also co-labels with proliferating cell nuclear antigen (PCNA)^12^, but detailed mechanisms of such interactions are lacking.

Previously we have established that depletion of Ataxin-10 in HeLa cells leads to aberrant multinucleated cells, indicative of cytokinetic failure^13^. We also identified that a key mitotic kinase, Polo-like kinase 1 (Plk1), coimmunoprecipitates (coIPs) with and phosphorylates Ataxin-10 at S77 and T82, which influences Ataxin-10's proteasome-dependent degradation and also cytokinesis. Cytokinesis is the final stage of cell division that generates two daughter cells^14^,^15^. During anaphase, the central spindles are compacted into the midbody as the cleavage furrow ingresses. In the spindle midzone and the midbody, embed a myriad of proteins, including Plk1and Aurora B^15^.

Here we further identify Aurora B as one of Ataxin-10's interacting partners. Aurora B is a critical component of the chromosome passenger complex, which orchestrates key events of the mitotic process, including chromosome-microtubule attachment, spindle assembly checkpoint activation, cytokinesis and eventual abscission^16^,^17^. We show that both endogenous and Aurora B-phosphorylated Ataxin-10 localizes to the midbody. In addition, the phospho-deficient S12A mutant fails to restore the cytokinesis defects caused by Ataxin-10 depletion and attenuates interaction between Ataxin-10 and Plk1. Our results thus delineate the role of Aurora B and Plk1 in regulating Ataxin-10 in cytokinesis.

## Results

### Ataxin-10 directly interacts with Aurora B

**Figure 1:**
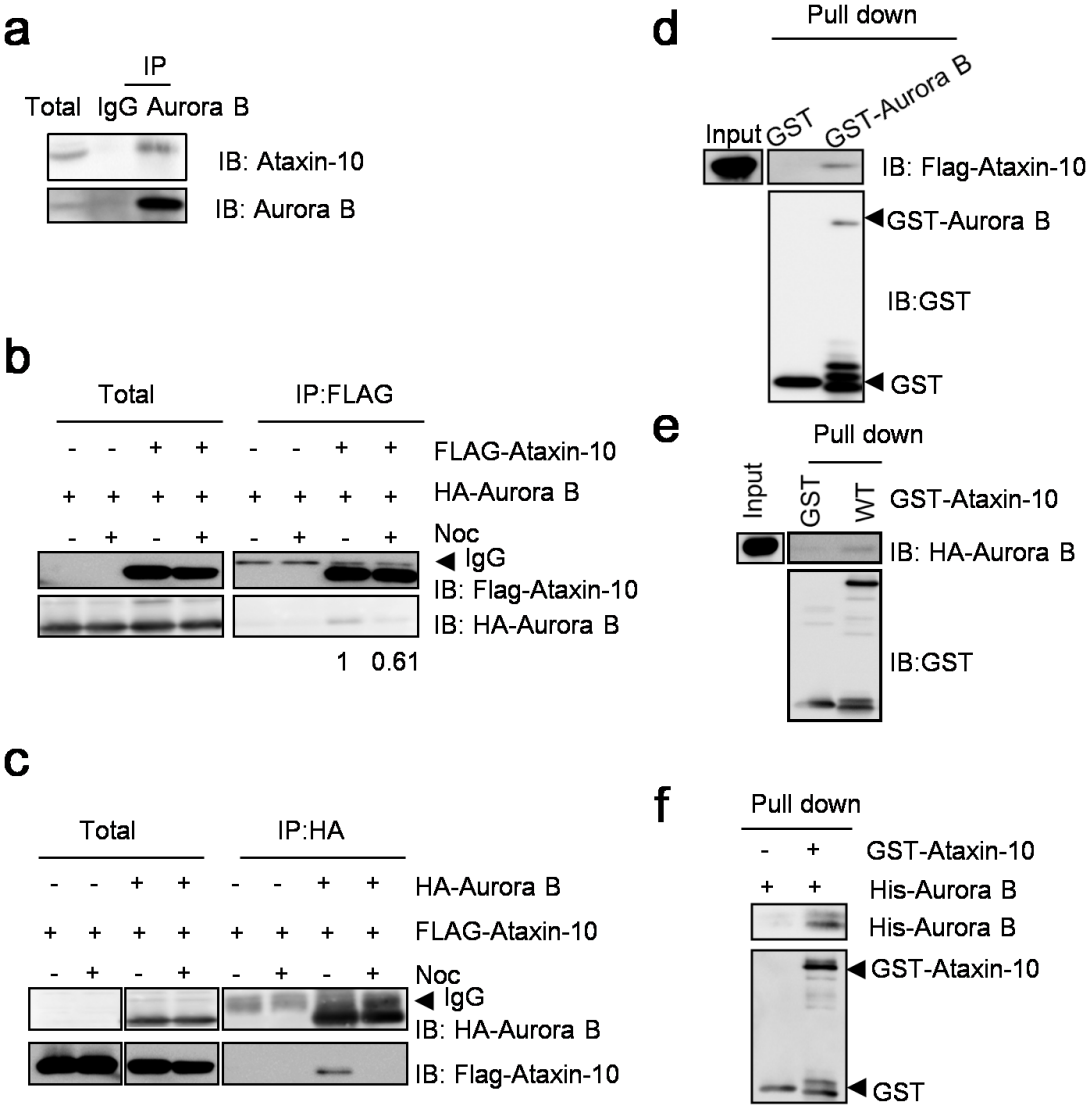
Interaction between Aurora B and Ataxin-10. (a) Co-IP between endogenous Ataxin-10 and Aurora B. Aurora B immunoprecipitates were blotted with anti-Ataxin-10 antibodies. IgG immunoprecipitates were negative controls. Two anti-Aurora B antibodies were used for IB: Bethyl (A300–431A) and Santa Cruz (SC-25426). (b and c) Co-IP between FLAG-Ataxin-10 and HA-Aurora B. HeLa cells were transfected with vector or FLAG-Ataxin-10, HA-Aurora B constructs, treated or untreated with Noc. (d and e) Bacterially expressed GST-Aurora B was incubated with lysates from HeLa cells transfected with FLAG-Ataxin-10 (d), or GST-Ataxin-10 incubated with HA-Aurora B (e). (f) GST-Ataxin-10 and His-Aurora B were both bacterially expressed, incubated together and were subject to pull-down assays. Uncropped images of blots were shown in supplemental Figure S1.

During our previous investigation of the interaction between Ataxin-10 and Plk1^15^, we inadvertently identified the association between endogenous Ataxin-10 and Aurora B (Fig. 1A). To exclude the possibility that the interaction was caused by fortuitous coIP by the antibodies we used, we constructed FLAG-Ataxin-10 and HA-Aurora B plasmids and transfected them into HeLa cells. CoIP experiments using anti-FLAG antibodies showed that overproduced FLAG-Ataxin-10 coIPed with HA-Aurora B (Fig. 1B). Reciprocally, HA-Aurora B coIPed with FLAG-Ataxin-10 (Fig. 1C). When we used nocodazol (Noc) to enrich mitotic cells, the interaction weakened markedly (Fig. 1 B–C), suggesting that the association was somewhat dampened during mitosis.

To assess whether Aurora B and Ataxin-10 interacted directly, we first bacterially expressed and purified GST-Aurora B, and used it to pull-down transfected FLAG-Ataxin-10 in HeLa cells, and found that recombinant GST-Aurora B retrieved FLAG-Ataxin-10 (Fig. 1D). Reciprocally, we used bacterially purified GST-Ataxin-10 to pull-down transfected HA-Aurora B, and again the association was observed (Fig. 1E). Lastly, we incubated GST-Ataxin-10 in conjunction with His-Aurora B, and GST-Ataxin-10 pull-downed His-Aurora B (Fig. 1F). Thus, these data suggested that Aurora B directly associated with Ataxin-10.

### Aurora B-dependent phosphorylation of Ataxin-10 at S12

**Figure 2:**
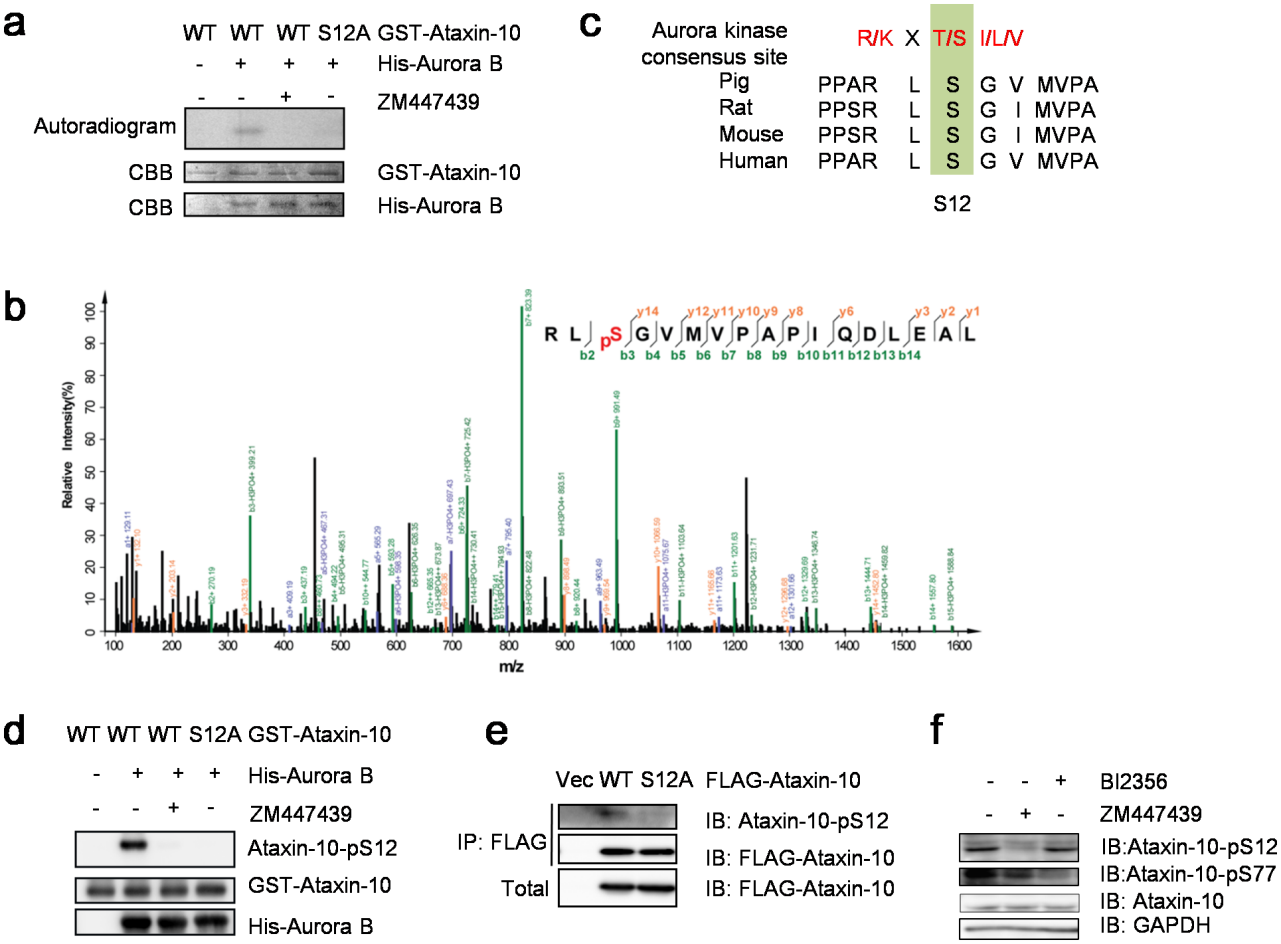
Identification of phosphorylation sites in the Ataxin-10 proteins. (a) GST-Ataxin-10-WT, S12A, and His-Aurora B were purified from *E. coli* cells and incubated in kinase buffers containing ^32^P-ATP with or without ZM. Coomassie blue (CBB) staining showed input Ataxin-10 proteins, and autoradiography detected phosphorylated GST-Ataxin-10. (b) Mass spectrum identified an Ataxin-10 peptide phosphorylated at S12. From this collision-induced dissociation spectrum, a phosphorylated peptide RL(pS)GVMVPAPIQDLEAL of Ataxin-10 was identified following incubation with Aurora B in an IVK reaction. “b” and “y” ion series represented fragment ions containing the N- and C-termini of the peptide, respectively. (c) A comparison of S12 and Aurora B phosphorylation consensus motif. The residues in red fit the consensus motif. (d) Recombinant GST-tagged wild-type Ataxin-10 or the S12A mutant were purified from *E. coli*, and incubated with recombinant His-Aurora B, or His-Aurora B with ZM. The samples were then analyzed by Western blot with anti-pS12 antibodies. (e) FLAG-tagged wild-type Ataxin-10 or the S12A mutant were transfected into HeLa cells and the cell lysates were subject to IP and IB analysis using the antibodies indicated. (f) HeLa cells were synchronized in cytokinetic phase as described in Methods and treated with 4 mM ZM447439 (Aurora kinase inhibitor) or 1 μm BI2356 (Plk inhibitor) for 2 hrs, then blotted with antibodies towards Ataxin-10, pS12 or pS77. Uncropped images of blots were shown in supplemental Figure S1.

To investigate whether Aurora B regulates Ataxin-10 through phosphorylation, we used in vitro kinase (IVK) assays with recombinant GST-Ataxin-10 and His-Aurora B. Aurora B phosphorylated Ataxin-10 efficiently, and the phosphorylation signal was reduced upon Aurora B inhibitor ZM447439 (ZM) treatment (Fig. 2A). The IVK products were then analyzed by liquid chromatography-tandem mass spectrometry (LC-MS/MS), and only one phosphorylation site, S12, was identified (Fig. 2B). To confirm that S12 was the in vitro phosphorylation site, we mutated it into phospho-deficient Ala and generated the S12A mutant. When incubated with His-Aurora B, the resultant GST-Ataxin-10-S12A phosphorylation signal decreased significantly (Fig. 2A).The amino acid sequence surrounding the S12 residue was also examined for Aurora kinase consensus motif, and a minimum consensus for Aurora B-dependent phosphorylation was identified (Fig. 2C), as Aurora B preferentially phosphorylates its substrates at serines or threonines preceded by positively charged residues^18^. In addition, S12 is also conserved among all Ataxin-10 mammalian homologues (Fig. 2C). These results suggest that S12 could be the major phosphorylation site.

To validate whether Ataxin-10 is a substrate of Aurora B in vivo, we raised a polyclonal phospho-specific antibody, Ataxin-10-pS12, as described in Methods. Indeed, Ataxin-10-pS12 was readily detected in the IVK assays using wild-type (WT) Ataxin-10, but not in the Ataxin-10-S12A assays, or in samples treated with ZM (Fig. 2D). Furthermore, when FLAG-Ataxin-10 and S12A were transfected into HeLa cells, Ataxin-10-pS12 was detected in the WT extracts, but the signal was reduced in the S12A extracts (Fig. 2E), suggesting that phosphorylation at S12 also occurred in vivo. Moreover, we used ZM to confirm whether phosphorylation at S12 was dependent on Aurora B. HeLa cells were enriched at cytokinetic stage as described in Methods, and treated with ZM or another kinase inhibitor BI2356 (BI), which targets Plk1. ZM treatment indeed abolished the pS12 signal, while BI did not (Fig. 2F), suggesting that pS12 of Ataxin-10 was Aurora B-dependent.

### Ataxin-10-pS12 is associated with the midbody

**Figure 3:**
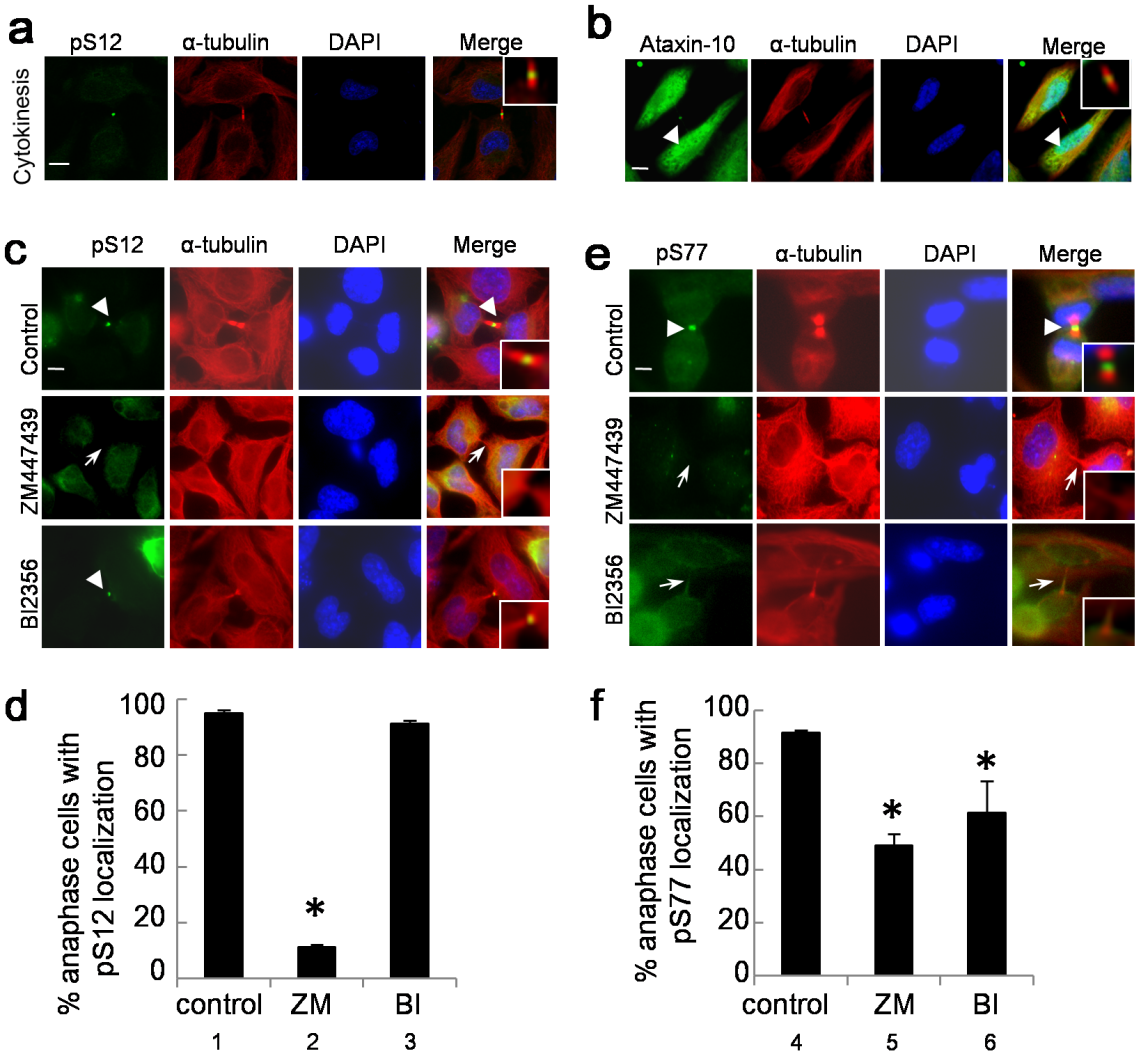
Ataxin-10-pS12 associates with the midbody. (a–b) HeLa cells were stained with anti-pS12 (a) (green) or anti-Ataxin-10 antibodies (b), anti-α-tubulin antibodies (red) and DAPI. The arrow head pointed to the midbody. (c) The midbody-specific pS12 staining was dependent on Aurora B, but not on Plk1. HeLa cells were enriched in cytokinetic phase, then treated with 1 μm BI or 4 mM ZM, before staining with pS12 antibodies. Open arrowheads indicated staining at the midbody, while open arrows indicated absence of staining at the midbody. (d) The results were quantitated. Data represent mean ± SD of three independent experiments, and more than 200 cells were counted in each experiment. Asterisks indicate significant differences from control cells, as determined by t-test (p_1–2_ = 0.001). Midbodies in a–d are maginifed and shown in insets. (e) The midbody-specific pS77 staining was dependent on Plk1 and Aurora B. Cells treated the same way as in (c), and then stained with pS77 antibodies, and quantitated in (f). The p values are p_4–5_ = 0.004, p_4–6_ = 0.0045. Scale bar, 10 μm.

To further assess the temporal and spatial regulation of pS12, we used the phospho-antibody to perform cytological experiments. Immunostaining assays were carried out combined with anti-α-tubulin antibodies to indicate the cell cycle stages. The results unveiled that pS12 displayed specific staining signals around the midbody during telophase (Fig.3A). The midbody localization pattern of pS12 reminded us to examine whether the endogenous protein localizes to the midbody. HeLa cells were enriched in cytokinetic stage and stained with anti-Ataxin-10 antibodies. And indeed, Ataxin-10 localized to the midbody (Fig. 3B). Additionally, it localized in the cytoplasm and nucleus, as previously reported^7^. We further treated the cells with BI, and Ataxin-10 remains at the midbody (supplemental Figure S2), suggesting that Plk1 activity is not required for midbody localization of Ataxin-10. These data suggest that both endogenous and S12 phosphorylated Ataxin-10 localized to the midbody.

### Association between Ataxin-10 and Plk1 is regulated by Aurora B-dependent phosphorylation

Given that we previously showed that Ataxin-10 was phosphorylated by Plk1^13^, we sought to delineate the relationship of Aurora B and Plk1 in terms of regulating Ataxin-10 in cytokinesis by cytology. HeLa cells treated with ZM or BI were stained with pS12 together with anti-α-tubulin antibodies, and we examined the cells at the midbody stage. As evidenced by Fig. 3C–D, ZM treatment significantly dampened the midbody-specific signal of pS12 from 91% to 11%, while BI treatment had relative little effect. The same experiment was carried out with pS77. Both ZM and BI resulted in significant decrease of pS77 localization at the midbody (Fig. 3E–F).

**Figure 4:**
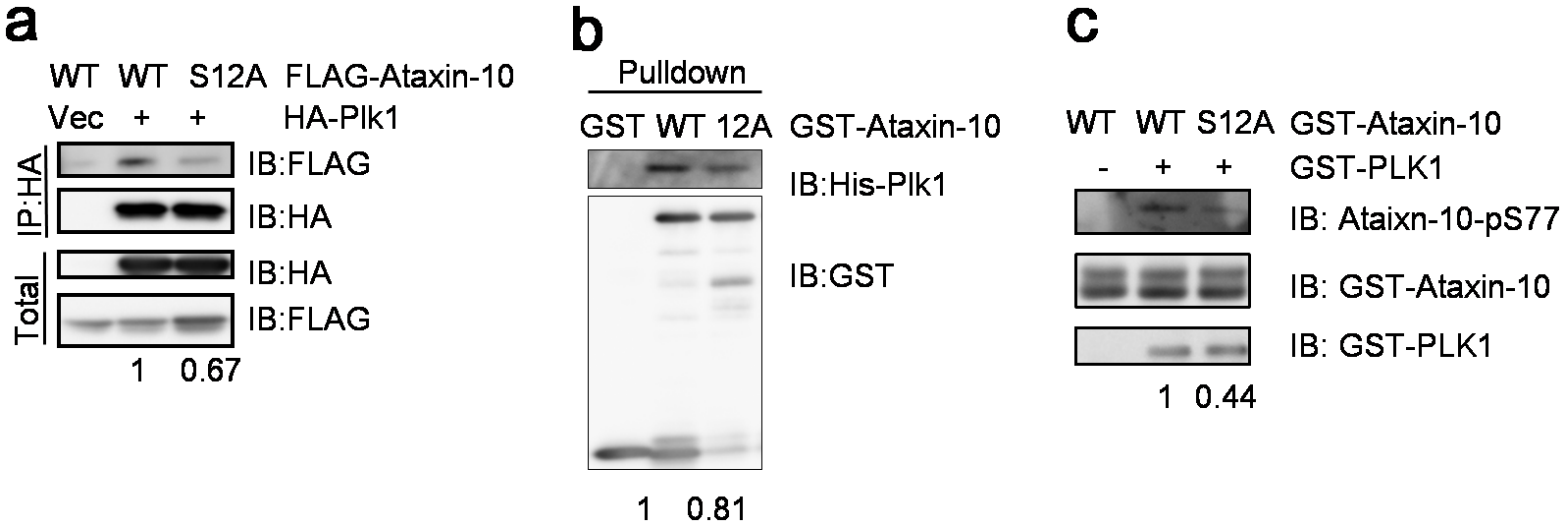
Association between Ataxin-10 and Plk1 is regulated by Aurora B-dependent phosphorylation. (a) HeLa cells were transfected with vector or FLAG-Ataxin-10-WT, or FLAG-Ataxin-10-S12A and HA-Plk1 constructs. (b) GST-Ataxin-10 and GST-Ataxin-10-S12A were incubated with His-Plk1, and were subject to pull-down assays. (c) Recombinant GST-tagged wild-type Ataxin-10 or the S12A mutant were incubated with GST-Plk1 and subject to IVK assay, then blotted with antibodies indicated. Numbers on the bottom of the Western blot indicate quantification of protein levels. Uncropped images of blots were shown in supplemental Figure S1.

Furthermore, biochemical essays were carried out. We transfected FLAG-Ataxin-10-WT or S12A together with HA-Plk1 into HeLa cells. CoIP experiments demonstrated that the interaction between S12A and Plk1 decreased ~30% compared with wild type (Fig. 4A). We also used GST-Ataxin-10-WT and GST-Ataxin-10-S12A to pull-down His-Plk1, and the interaction reduced ~20% (Fig. 4B). We reasoned that if S12A showed decreased interaction with Plk1, then Plk1 would phosphorylate Ataxin-10-S12A less efficiently. And indeed it was the case. GST-tagged WT Ataxin-10 or the S12A mutant were incubated with GST-Plk1 and subject to IVK assays, and immunoblotting demonstrated that there is ~50% reduction of phosphorylation at S77 in the S12A mutant (Fig. 4C), suggesting that phosphorylation at S77 was partially dependent on pS12. We also tested whether the interaction between Ataxin-10 and Plk1 depends on the kinase activity of Plk1 (supplemental Figure S3). BI treatment did not alter the coIP between Plk1 and Ataxin-10. We conclude that Aurora B promotes the association between Ataxin-10 and Plk1 by S12 phosphorylation.

### Wild-type Ataxin-10, but not the Ataxin-10-12A mutant, partially rescues the cytokinesis defects

**Figure 5:**
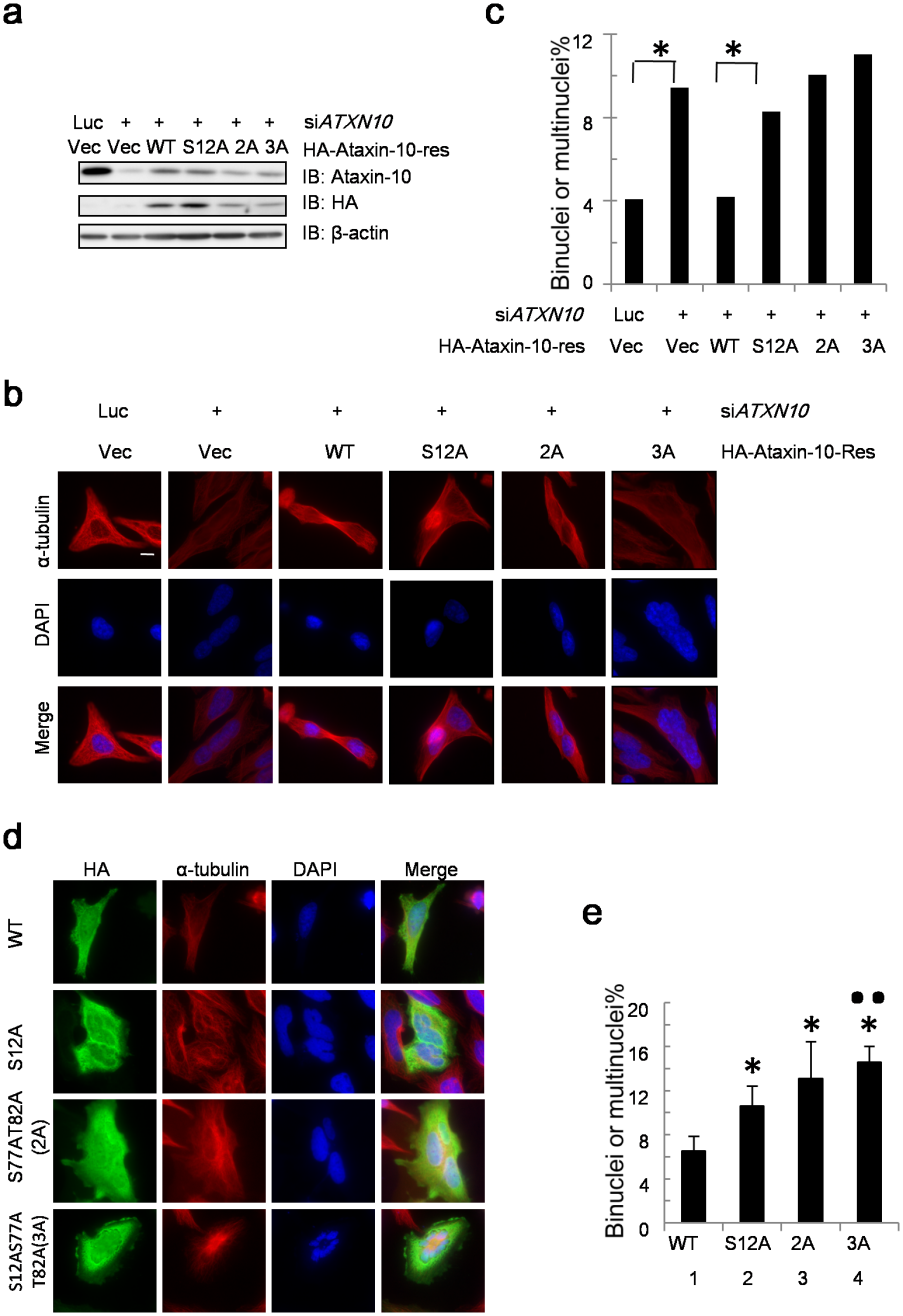
S12 is essential for cytokinesis. (a) HeLa cells were stably transfected with HA vector, HA-*ATXN10-res* and HA-*ATXN10-12A-res*, S77AT82A (2A) *-res*, S12AS77AT82A(3A) *-res* plasmids, and then treated with control siRNA or si*ATXN10*, as indicated. The lysates were collected 48 hrs later and subject to IB using the antibodies indicated. (b) the same cells in (a) were analyzed by immunofluorescence for multinucleated cells and the data were quantified. The data are representative of two independent results. More than 100 cells were counted in each sample, and asterisks indicate significant differences as determined by χ^2^ test. (d) HeLa cells ectopically expressing HA-Ataxin-10, S12A, 2A, or 3A mutants were costained with anti-HA and anti-α-tubulin antibodies. Binucleated or multinucleated cells were scored. Scale bar, 10 μm. (e) The histogram shows the mean ± SD from three independent experiments. More than 100 HA-positive cells were counted in each experiment. Asterisks indicate significant differences from wild type (p < 0.05); double dots indicate significant differences from S12A (p < 0.05), as determined by t-test (p_1–2_ = 0.007, p_1–3_ = 0.039, p_1–4_ = 0.00017, p_2–3_ = 0.129, p_2–4_ = 0.008, p_3–4_ = 0.39). Uncropped images of blots were shown in supplemental Figure S1.

Since Aurora B kinase is involved in multiple steps of the mitotic pathway, we examined whether the phosphorylation of Ataxin-10 by Aurora B contributes to cytokinesis. We performed rescue assays using HA-*ATXN10-res* and HA-*ATXN10-S12A-res* plasmids. These plasmids were transformed into HeLa cells and the resultant stable transfectants were treated with si*ATXN10*, as indicated in Fig. 5A. Endogenous Ataxin-10 was attenuated substantially (decreased ~85%), while the exogenous HA-Ataxin-10-res and HA-Ataxin-10-12A-res were stably expressed, as shown by Western blot (Fig. 5A). These cells were then examined by immunofluorescence for multinucleated cells. Re-expression of HA-*ATXN10-res* partially restored the cytokinesis defects induced by si*ATXN10* (Fig. 5B). The HA-*ATXN10-12A-res* plasmids, however, did not efficiently restore the defects. The results were then subject to χ^2^ statistical analysis (Fig.5C). Since we have shown previously that Plk1 phosphorylates Ataxin-10 at S77 and T82^13^, and that both residues are important for cytokinesis, we constructed the S12AS77AT82A (3A) mutant, and compared the cytokinesis defects. HA-*ATXN10-2A-res* and HA-*ATXN10-3A-res* plasmids were also transfected, and the cytokinetic phenotypes were examined. There is no significant difference among 12A, 2A, or 3A rescue plasmids (Fig.5B–C). Thus, the rescue experiments indicate that S12 is essential for Ataxin-10 to participate in cytokinesis.

To address the question whether the S12 site is functional in vivo, we examined the cytological defects in cells expressing phospho-deficient *ATXN10*-*S12A* plasmids. About 6% control cells contained two or more nuclei. But when the S12A mutant was expressed, about 11% cells were multinucleated (Fig. 5D–E), which was statistically different by t-test, suggesting that S12 is essential for Ataxin-10's role in cytokinesis.

We also compared the cytokinesis defects with the 3A mutant. While ~13% of cells transfected with S77AT82A showed cytokinesis defects, the 3A mutant had ~14%, which is not statistically significant (Fig. 5D–E). When we compared cells transfected with S12A and 3A, ~11% and 14% showed cytokinetic defects, respectively. They were also significantly different from each other (Fig. 5E). Our results suggest that Aurora B functions epistatic to Plk1 in terms of Ataxin-10 role in cytokinesis.

## Discussion

Here we identified Aurora B as one of Ataxin-10's binding proteins, following our previous work of the interaction between Plk1 and Ataxin-10^19^. Our results indicate that Aurora B functions upstream of Plk1 in regulating Ataxin-10 in cytokinesis.

Our findings suggest that Aurora B phosphorylates Ataxin-10 at S12, which colocalizes with the midbody, and that Aurora B-dependent phosphorylation of pS12 acts epistatic to Plk1-dependent phosphorylation of pS77. We think that pS12 promotes Ataxin-10 and Plk1's interaction to participate in cytokinesis, evidence of which includes: initially, BI has little effect on pS12 localization on midbody, but ZM significantly affects pS77 localization (Fig. 3C–F); additionally, S12A shows decreased interaction with Plk1 either in coIP or pull-down assays (Fig. 4A–C).

Our data also suggest that there might be other factors upstream of Plk1 in regulating Ataxin-10 in cytokinesis. First, S12A attenuated but did not completely abolish interaction with Plk1 (Fig. 4); second, the overproduction assay shows that there are statistical differences between the percentage of cells bearing cytokinetic defects between S12A and 3A rescue plasmids (Fig. 5). We envision that an unidentified mechanism also regulates Ataxin-10 and Plk1 interaction, which could be a third kinase or another form of modification. In fact, Ataxin-10 associates with OGT^10^,^11^, which catalyzes protein *O*-GlcNAcylation. However, it remains elusive whether Ataxin-10 itself is subject to *O*-GlcNAcylation. It is a possible scenario that glycosylation affects Ataxin-10's interaction with Plk1.

The interaction between Plk1 and its substrates are subject to multiple levels of regulation. As Plk1 contains a kinase domain and a conserved polo-box domain (PBD), a common theme has been a priming kinase, usually CDK1, first phosphorylates the substrates at a conserved S/T-P site, allowing phosphorylated substrates to dock to the PBD domain of Plk1, then the catalytic domain of Plk1 further phosphorylates the substrates^20^,^21^. This mechanism has been established by various studies, such as the case for MYPT1^22^ and CAP-D2 subunit of condensin II^13^, among many others. Our data suggest an alternative model, in which Aurora B first phosphorylates the potential substrate, thus increasing its association with Plk1. Consistent with our results, Wu et al^23^ identified that Aurora B phosphorylates myosin II-interacting guanine nucleotide exchange factor (MyoGEF) to promote the binding of Plk1 to MyoGEF at the central spindle. Thus, further studies are needed to examine whether this mechanism is widely adopted.

Plk1 is activated in G_2_ by Aurora A kinase, which is essential to recover DNA damage-induced cell cycle arrest^24^,^25^. On the other hand, Aurora B and INCENP, a regulatory protein of Aurora B activity, are essential for Plk1 activation at centromeres and kinetochores in *Drosophila* and cultured cells^26^. How Plk1 is activated during cytokinesis is still a puzzle^16^. Our model suggests that cytokinetic substrates of Plk1 might be regulated prior to associating with Plk1, thus adding another layer of complexity.

## Methods

### Cell culture, antibodies and plasmids

HeLa cells were purchased from ATCC. Cell culture, anti-Ataxin-10 antibodies, *ATXN10* plasmids were described before^19^. Antibodies against phospho-Ser77 (pS77-Ab) and phospho-Ser12 (pS12-Ab) were raised in rabbits using the sequence of CNLAS(pS)LQLIT and PPARL(pS)GVMVC, respectively. The antibodies were manufactured by Beijing B&M Biotech Co., Ltd. A*TXN10-S12A* and *ATXN10-S12AS77AT82A(3A)* mutants were generated using specific primers (sequences available upon request) following the manufacturer's instructions (QuickChange II, Stratagene).

### Transfections

HeLa cells were transfected twice with a 24-h interval using Oligofectamine (Invitrogen) according to the manufacturer's instructions. Transfectants were used for further experiments 24 hours after the second transfection. All small interfering RNAs (siRNA) oligonucleotides duplexes were purchased from Dharmacon. The control siRNA oligonucleotides duplex was CONTROLsi: CGUACGCGGAAUACUUCGAdTdT. *ATXN10*siRNA oligonucleotides duplexes were: *ATXN10*-si2: CAACAUUGCCUCACGGAAU. HA-*ATXN-10*-res plasmids were constructed targeting *ATXN10* siRNA by site-directed mutagenesis with primer sequences: CAATATAGCGAGTCGCAAT.

For plasmid transfection, cells were seeded at 50-60% confluence/10 cm^2^ petri dish and transfected with 7.5 μg of plasmid DNA using FuGene 6 according to the manufacturer's instructions for immunoprecipitation (IP) experiments, or 1.5 μg to six-well plates for immunofluorescence (IF) experiments.

### Indirect immunofluorescence staining and cell culture synchronization

Indirect immunofluorescence staining was performed as described before^19^. Dilutions of primary antibodies were 1:1,000 for mouse anti-α-tubulin. Cell nuclei were stained with DAPI. Cell culture synchronization for IF and IP was performed as described^27^. Briefly, cell cultures were first blocked by double thymidine. Nine hours after release from the second thymidine block, 4 mM ZM447439 (Aurora kinase inhibitor) or 1 μm BI2356 (Plk1 inhibitor) were added. Two hours later, cells were harvested.

### Immunoblotting and IP

Immunoblotting and IP experiments were performed as described before^19^. Nocodazole was used at 100 ng/ml for 16 hours. The following primary antibodies were used for immunoblotting: anti-Ataxin-10, anti-Aurora B, anti-β-actin, anti-HA and anti-FLAG M2 (Sigma). Peroxidase-conjugated secondary antibodies were from JacksonImmuno Research. Blotted proteins were visualized using the ECL detection system (Amersham). Signals were detected by a LAS-4000, and analyzed using Multi Gauge (Fujifilm).

### In vitro kinase assay

Aurora B in vitro kinase assay was performed as previously described^28^. Briefly, recombinant Aurora B kinase was incubated with purified GST-Ataxin-10 with 10 mM HEPES (pH 7.5), 50 mM NaCl, 2 mM MgCl_2_, 1 mM Dithiothreitol, 1 mM EGTA, 0.1 mM ATP or 1 μCi of γ-[^32^P]ATP. After 20 min at 30°C, reactions were stopped by the sample buffer. Protein samples were separated by SDS-PAGE and phosphate incorporation was determined by PhosphorImager.

### LC-MS/MS analysis

After an IVK assay, the proteins were precipitated with 25%TCA, washed with 500 μl cold acetone twice, air dried and resuspended in 8 M urea, 100 mM Tris (pH 8.5). After reduction (5 mM TCEP at room temperature for 20 min) and alkylation (10 mM iodoacetamide at room temperature for 15 min), the samples were diluted to 2 M urea with 100 mM Tris (pH 8.5), and each divided into four aliquots for Trypsin, Asp-N, Glu-C or Elastase digestion (1:50 enzyme: substrate, 25°C for Glu-C and 37°C for others, overnight). The digestions were quenched with 5% formic acid and pooled. The peptides were separated on a C18 resin (Luna 3 μm, 100 Å), the LC-MS/MS analysis was performed on an Easy-nLC 1000 UPLC (Thermo Fisher Scientific) coupled to a Q Exactive mass spectrometer (ThermoFisher Scientific). Peptides were loaded on a pre-column (75 μm ID, 8 cm long, packed with ODS-AQ 12 nm-10 μm beads from YMC Co., Ltd) and separated on an analytical column (75 μm ID, 11 cm long, packed with Luna C18 3 μm 100 Å resin from Phenomenex) using an acetonitrile gradient from 0–30% in 55 min and 30–80% in another 10 min at a flow rate of 300 nl/min. Spectra were acquired in a data-dependent mode: the 10 most intense precursor ions from each full scan (Resolution 70,000) were isolated for HCD MS2 (Resolution 17,500) at NEC 27 with a dynamic exclusion time of 60 s. Precursors with 1+ or unassigned charge states were excluded. For peptide identification, the MS2 spectra were searched against an EPI-IPI human database (forward and reversed sequences) using Prolucid with 50 ppm mass accuracy for both precursor and fragment ions, with carbamidomethylation on cysteine as fixed modification and phosphorylation (79.9663) on serine, threonine, or tyrosine as differential modification^29^. Search results were filtered using DTASelect 2.0 with 7 ppm mass accuracy for precursor mass and a 5% FDR cutoff at the spectral level^30^. The phosphopeptide spectrum was annotated using pLabel, requiring 20 ppm mass accuracy and 1% intensity threshold for fragment ions^31^.

## Author Contributions

J.Li. wrote the manuscript; J.Li., M.-Q.D. and X.X. designed the project and analyzed the data; J.T., C.T., Y.D., Z.L., Q.G., Z.X., J.W., W.H. and J.Liao. prepared the figures. All authors reviewed the manuscript.

## Supplementary Material

Supplementary Information Supplemental information
