# Supplementary material for: Aurora B-dependent phosphorylation of Ataxin-10 promotes the interaction between Ataxin-10 and Plk1 in cytokinesis
Source: Sci Rep. 2015 Feb 10;5:8360. doi: 10.1038/srep08360 (PMC4322367; doi:10.1038/srep08360)
Supplement: Supplementary Information — Supplemental information [file srep08360-s1.pdf]

Aurora B-dependent phosphorylation of Ataxin-10 promotes the interaction between Ataxin-10 and Plk1 in cytokinesis

Jie Tian[1], Chuan Tian[1], Yuehe Ding[2], Zhi Li[1], Qizhi Geng[1], Zhikai Xiahou[1], Jue Wang[1], Wenya Hou[1], Ji Liao[1], Meng-Qiu Dong[2], Xingzhi Xu[1, §] and Jing Li[1, §]

Supplemental information

Figure S1 Full scans of immunoblots in this study.

Figure S2 Plk1 activity is not required for the midbody localization of Ataxin-10. HeLa cells were treated with BI or left untreated, then stained with anti-Ataxin-10, anti- α -tubulin antibodies and DAPI. Midbodies are maginifed and shown in insets.

Figure S3 Plk1 activity is not required for the interaction between Plk1 and Ataxin-10. Co-IP between FLAG-Plk1 and HA-Ataxin-10. HeLa cells were transfected with vector or HA-Ataxin-10, FLAG-Plk1 constructs, treated or untreated with BI.

Figure 1A

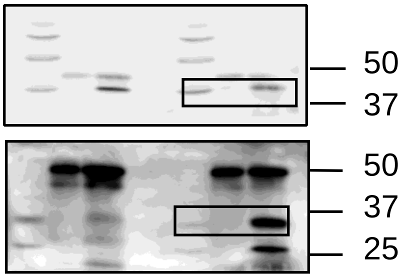

Figure 1B

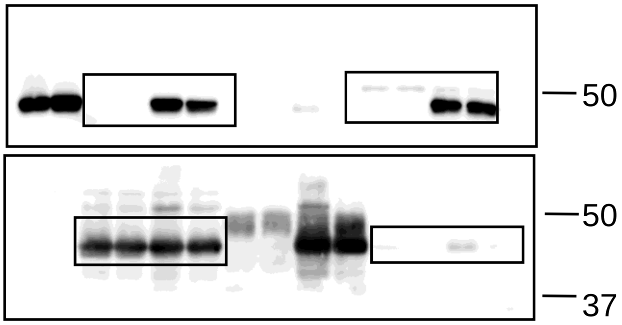

Figure 1C

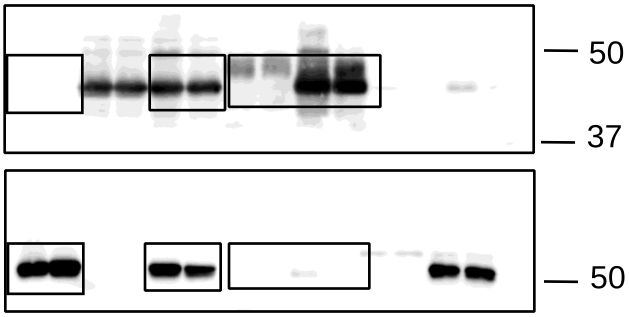

Figure 1D

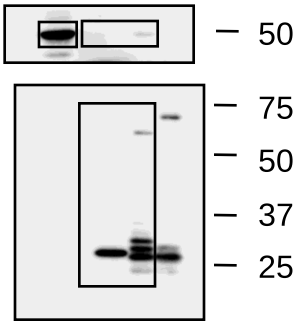

Figure 1E

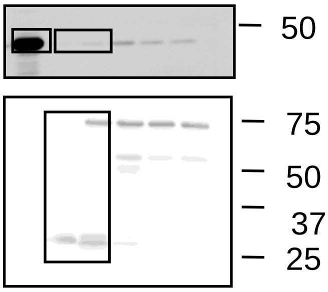

Figure 1F

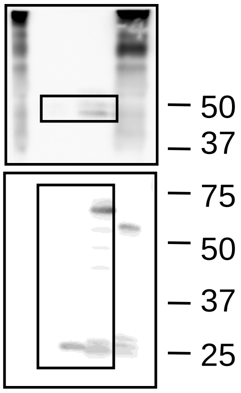

Figure 2A

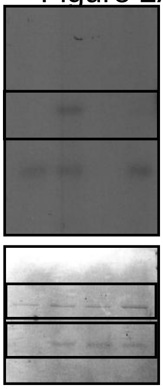

Figure 2D

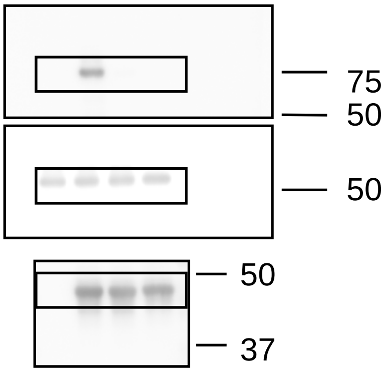

Figure 2E

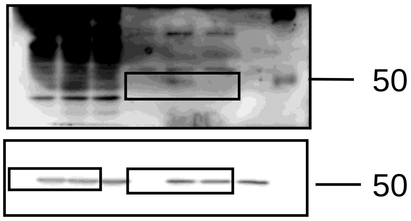

Figure 2F

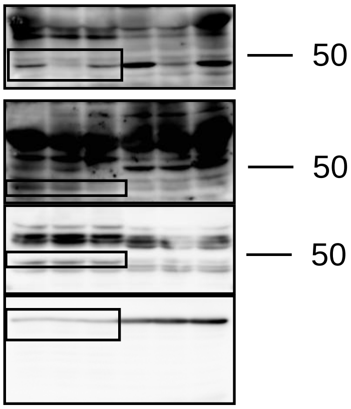

Figure 4A

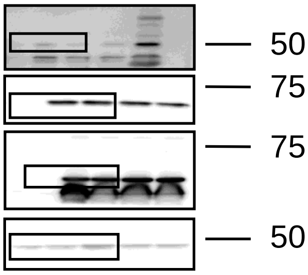

Figure 4B

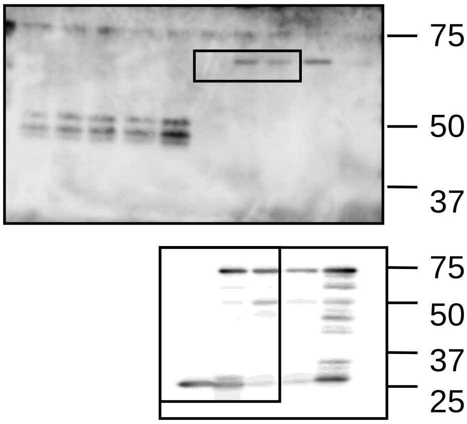

Figure 4C

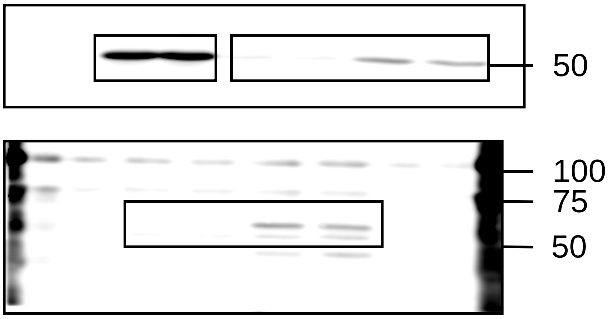

Figure 4D

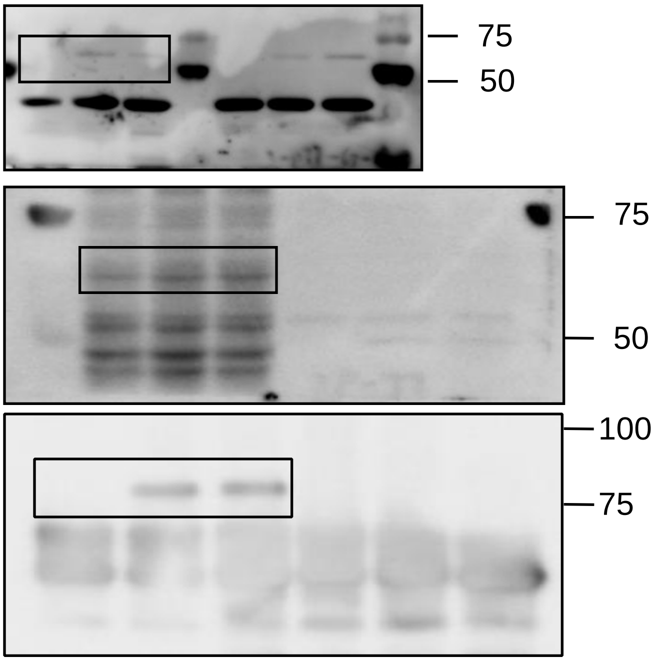

Figure 5A

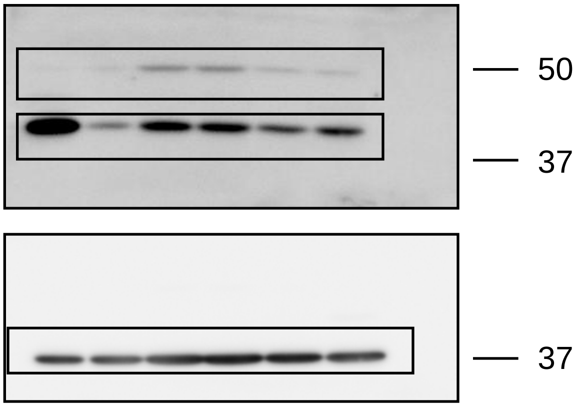

Figure_S1 Tian

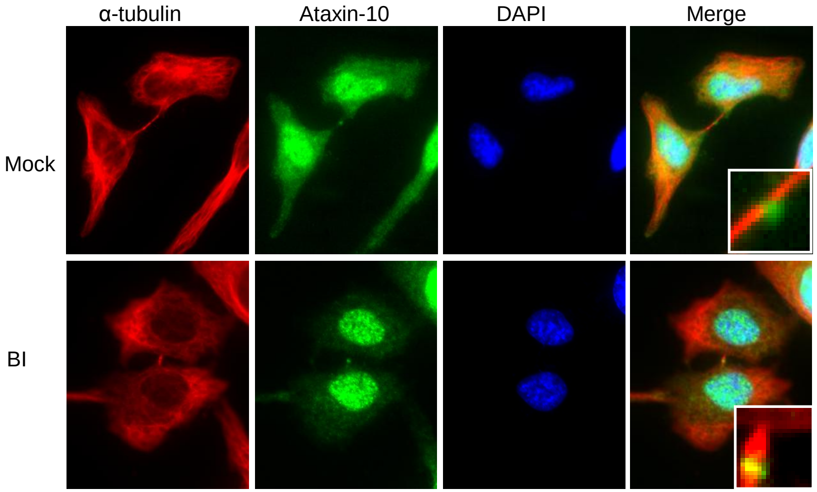

Figure_S2 Tian

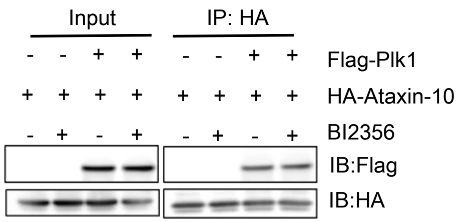

Figure_S3 Tian
